# A practical implementation of large transcriptomic data analysis to resolve cryptic species diversity problems in microbial eukaryotes

**DOI:** 10.1186/s12862-018-1283-1

**Published:** 2018-11-16

**Authors:** Yonas I. Tekle, Fiona C. Wood

**Affiliations:** Spelman College, 350 Spelman Lane Southwest, Atlanta, GA 30314 USA

**Keywords:** RNA-seq, Amoebozoa, DNA barcode, Bioinformatics, Cryptic species, Phylogenomics

## Abstract

**Background:**

Transcriptome sequencing has become a method of choice for evolutionary studies in microbial eukaryotes due to low cost and minimal sample requirements. Transcriptome data has been extensively used in phylogenomic studies to infer ancient evolutionary histories. However, its utility in studying cryptic species diversity is not well explored. An empirical investigation was conducted to test the applicability of transcriptome data in resolving two major types of discordances at lower taxonomic levels. These include cases where species have the same morphology but different genetics (cryptic species) and species of different morphologies but have the same genetics. We built a species comparison bioinformatic pipeline that takes into account the nature of transcriptome data in amoeboid microbes exemplifying such discordances.

**Result:**

Our analyses of known or suspected cryptic species yielded consistent results regardless of the methods of culturing, RNA collection or sequencing. Over 95% of the single copy genes analyzed in samples of the same species sequenced using different methods and cryptic species had intra- and interspecific divergences below 2%. Only a minority of groups (2.91–4.87%) had high distances exceeding 2% in these taxa, which was likely caused by low data quality. This pattern was also observed in suspected genetically similar species with different morphologies. Transcriptome data consistently delineated all taxa above species level, including cryptically diverse species. Using our approach we were able to resolve cryptic species problems, uncover misidentification and discover new species. We also identified several potential barcode markers with varying evolutionary rates that can be used in lineages with different evolutionary histories.

**Conclusion:**

Our findings demonstrate that transcriptome data is appropriate for understanding cryptic species diversity in microbial eukaryotes.

**Electronic supplementary material:**

The online version of this article (10.1186/s12862-018-1283-1) contains supplementary material, which is available to authorized users.

## Background

The vast majority of eukaryotic diversity is microbial, but many aspects of their behavior and biodiversity remain poorly understood [1–3]. Our understanding of the evolution of microbial eukaryotes is steadily increasing with analysis of molecular data [4–6]. However, microbial eukaryotes are generally undersampled in genome scale analyses, where most genome-scale studies have focused on model and medically important microbes [7–10]. More recent developments in high-throughput sequencing (HTS) techniques are allowing generation of large amounts of genetic data from non-model organisms through alternative (reduced genomic) approaches (e.g. transcriptomics, restriction site-associated DNA (RAD), metagenomics). The large amounts of genetic data generated from HTS of previously neglected microbial lineages are contributing to our understanding of the eukaryotic tree of life [11–14]. Despite the exponential growth of genetic data, the practical applications of HTS in studies such as cryptic species biodiversity has not been fully explored, and is limited to only a few genes or lineages [15–18].

The problem of cryptic species in taxonomy has been known since Linnaean time. The full extent of the challenges it posed to taxonomy and other related fields such as conservation biology, agriculture and diagnostic medicine was realized with the advent of molecular techniques [19–24]. Common manifestations of discordance between morphological and genetic data are observed when morphologically indistinguishable species have different genetic makeup, or vice versa [25]. In the first case, genetically distinct species, with divergences above the commonly defined species delimitation thresholds [26–28], appear similar or indiscernible at the gross morphology level. This is a common problem in microbes since their taxonomy has long suffered from plasticity and paucity of diagnostic morphological characters [2]. This type of discordance is a major impediment in biodiversity studies, as genetically distinct species are lumped together into one operational taxonomic unit (OTU). The second, less typical, case of discordance occurs when morphologically distinct lineages are genetically identical. This could result in overestimation of biodiversity by splitting the same species into different OTUs.

DNA sequencing of single or few markers, selected for their DNA barcode potential, has played an instrumental role in uncovering hidden diversity in living organisms [26, 29–31]. A mitochondrial gene, cytochrome oxidase 1 (COI), has been extensively used in species delimitations and resolving cryptic species diversity, mostly in animals [26] but also in some microbes [27]. However, more recent studies have revealed a number of limitations for its universal applicability, particularly in species boundary delimitations [32]. These include variation in rates of evolution in mitochondrial genes as well as other concerns related to patterns of inheritance, recombination and heteroplasmy (reviewed in [33]). Thus, species boundaries in some lineages cannot be determined with certainty using COI alone [33, 34]. The full impact of this problem in microbes is not well investigated. However, there are some examples in amoeboid microbes demonstrating that the commonly used DNA barcode markers (COI or ribosomal genes) do not always work [29, 35]. The limitations of mtDNA and the idiosyncratic nature of DNA barcoding have led many to use an integrative approach - combining multiple data sources such as morphology, behavior and ecology with genetic data [36, 37]. However, microbes are generally poorly characterized and pose a special challenge due to the limited and plastic nature of the observed morphological characters [25]. Additionally, some microbes either lack or have highly reduced mitochondria [38], severely limiting the applicability of mitochondrial genes as universal barcode markers.

Since genomes contain the history of an organism, an ideal solution to resolving cryptic species diversity is to analyze whole genome data. However, this endeavor is not feasible due to the associated high cost as well as limited understanding of microbial genomes. Low cost alternative HTS approaches are allowing the generation of tens of thousands of genes from under-sampled microbes [39]. While most of the studies using these techniques have focused in reconstructing ancient histories, studies focusing on recent or lower taxonomic scales, such as species delimitation, are slowly emerging. Recently, RAD sequencing, a method of semi-randomly subsampling portions of the genome for genetic variation, has been successfully used in species delimitation studies [40, 41]. However, RAD sequencing requires a prior knowledge of genome size and GC content to choose restriction enzyme targets, which limits its use in microbes without genome data. Transcriptome sequencing provides thousands of coding gene sequences from small amounts of starting material, including single cells, without prior knowledge of the genome. Transcriptome data has played a significant role in phylogenomics studies involving deep evolutionary divergences in eukaryotes (e.g. [14, 42]). However, its utility in studying cryptic species diversity is not well explored. Given the growing amount of transcriptome data in microbial eukaryotes, it is prudent to test its utility in understanding cryptic species biodiversity.

In this study we used transcriptome data to address cryptic species diversity problems in microbial eukaryotes. We developed a bioinformatics pipeline suited to handle transcriptome data for comparative study taking into account the nature of sequence quality and paralogy. We analyzed up to 30,000 transcripts (contigs) per sample in amoeboid microbes exemplifying the two major discordances of molecular and morphological data. These lineages including a thecamoebida isolate, *Cochliopodium* and *Endostelium* representing diverse groups within one of the major subclades (Discosea) of Amoebozoa. Our transcriptomic comparative analysis revealed a consistent pattern of inter- and intra-specific divergences among known or suspected cryptic species. We also identified several barcode markers with varying evolutionary rates that can be used in microbial eukaryotes. Our findings demonstrate the appropriateness of transcriptome data for cryptic species diversity studies.

## Results

### Probing the nature of transcriptome data using a bioinformatic pipeline

**Fig. 1:**
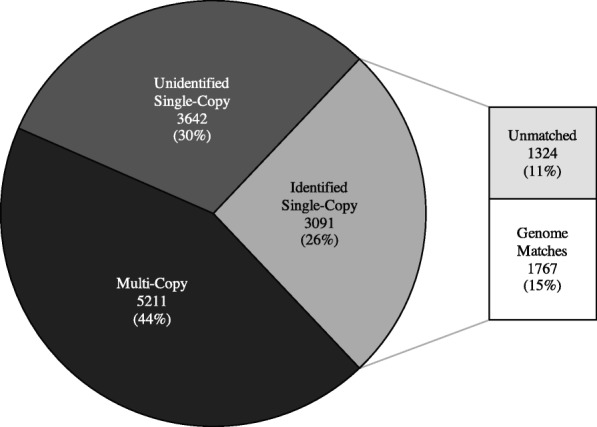
Proportion of all orthologous groups from the transcriptomes of *Cochliopodium pentatrifurcatum*, *C. minus*, and *C. minutoidum,* which were identified as multi-copy or paralogs (black), single-copy but unidentified (unknown genes) (medium grey), or single-copy and identified (matching to known eukaryotic genes) (light grey). The latter category is further divided into those that match single-copy ortholog groups identified by OrthoVenn between *Acanthamoeba castellanii* and *Dictyostelium discoideum* genomes (light grey) and those that did not match to any such group (white)

We built a bioinformatic pipeline for comparative analysis of genomic and transcriptomic data from multiple species (see Fig. 5). This pipeline was designed specifically to handle transcriptome data by taking into account the nature of sequence quality and paralogy (Wood and Tekle in prep.). The pipeline takes assembled contigs from different genome and transcriptome samples and finds orthologous groupings, then generates distance matrices for comparative purposes. Using the pipeline we were able to successfully match tens of thousands of contigs between isolates of the same and different species within various genera. The initial output of the pipeline classifies contigs, previously categorized as eukaryotic or unidentified genes via BLAST comparisons against a reference eukaryotic database, into putative ‘single’ or multi-copy matched groups. Many of the ‘single’ eukaryotic groups were further inferred to likely be single-copy based on matches to single-copy clusters found from clustering the genomes of *Dictyostelium discoideum* and *Acanthamoeba castellanii* using OrthoVenn [43] (Fig. 1). These genes generally performed better in the transcriptome-wide comparisons than their unidentified or multi-copy counterparts (Additional files 1, 2, 3, 4, 5 and 6: Figures S1–S6), deduced from lower overall divergences between transcriptome data of the same species generated by different studies (Table 1).

**Table 1:** Proportion of contig groups with distances > 2% in each transcriptome comparison

	All	Single-Copy Eukaryotic	Matches to Single-Copy Genome Clusters
*Cochliopodium* spp.			
^a^*C. pentatrifurcatum*	7.99%	4.87%	4.17%
^a^*C. minus* CCAP 1537/1A	5.89%	3.60%	2.91%
*C. pentatrifurcatum* vs. *C. minus* CCAP 1537/1A	9.31%	4.31%	3.95%
*C. pentatrifurcatum* vs. *C. minutoidum* CCAP 1537/7	99.96%	99.92%	100%
*Endostelium zonatum*			
PRA-191 YT10 vs. PRA-191 Kang	6.81%	4.77%	4.02%
PRA-191 YT10 vs. LINKS	99.84%	99.77%	99.82%
PRA-191 Kang vs. LINKS	99.83%	100%	100%
Thecamoebida isolates			
UK-YT1 vs. Thecamoebida RHP1–1	10.58%	5.56%	4.19%

^a^intraspecific comparison of transcriptome data from same species collected using different methods

**Fig. 2:**
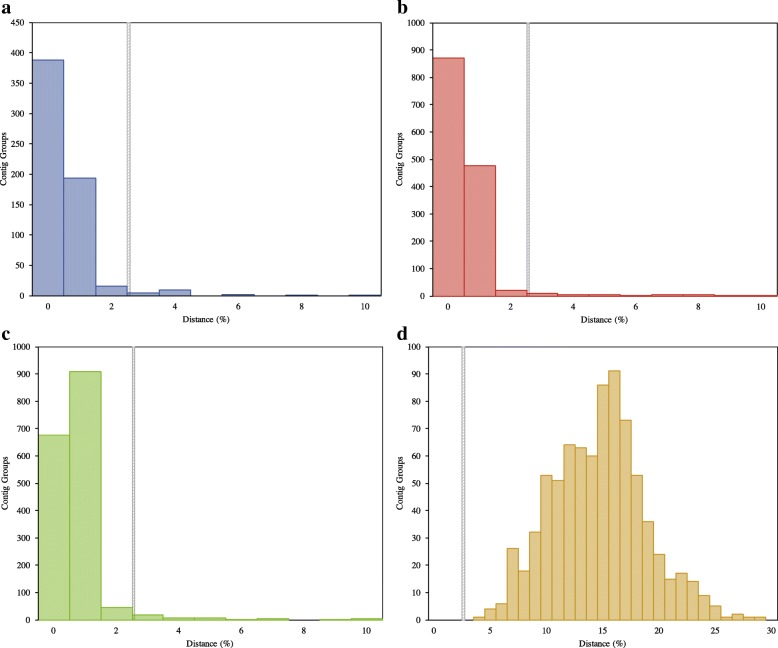
Histogram of average distances within genome-matching single-copy eukaryotic ortholog groups in the genus *Cochliopodium*. **a** *C. pentatrifurcatum* intraspecific variation. **b** *C. minus* intraspecific variation. **c** *C. pentatrifurcatum*-*C. minus* interspecific distance. **d** *C. pentatrifurcatum*-*C. minutoidum* interspecific distance

**Fig. 3:**
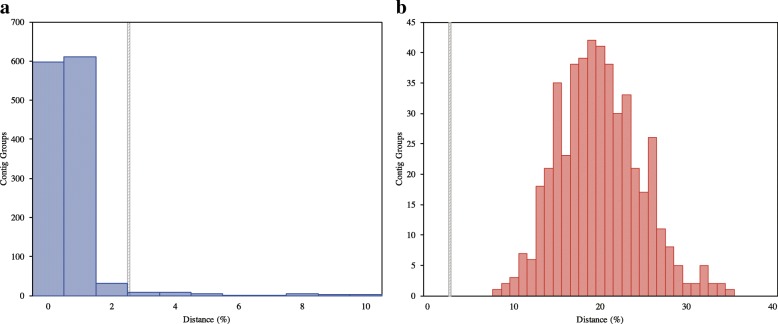
Histogram of average distances within genome-matching single-copy eukaryotic ortholog groups from the genus *Endostelium*. **a** *E. zonatum* PRA-191 transcriptomes sequenced in different labs [14, 46]. **b** *E. zonatum* PRA-191 sequenced by Kang et al. [14] vs. *‘E. zonatum’* LINKS sequenced by Kang et al. [14]

**Fig. 4:**
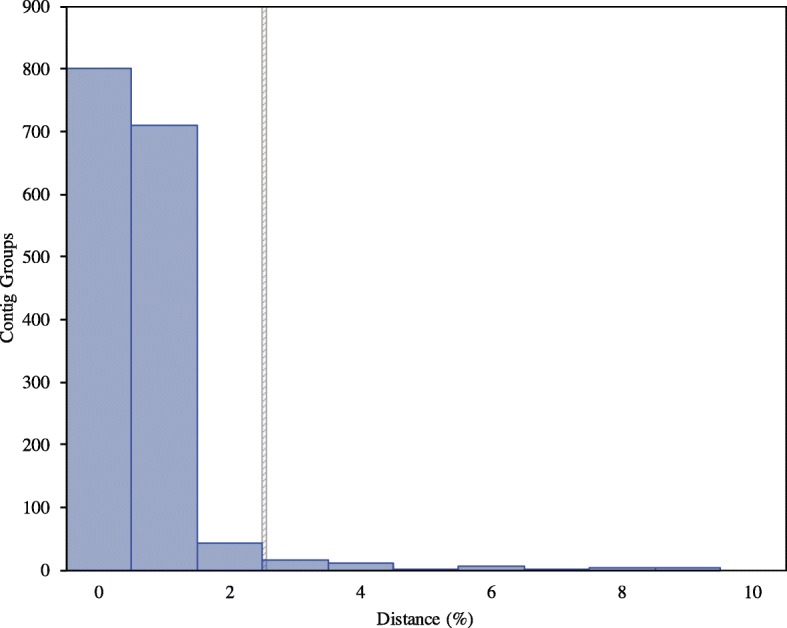
Histogram of average distances within genome-matching single-copy eukaryotic ortholog groups from comparison between an undescribed isolate UK-YT1 and Thecamoebida sp. RHP1–1

Our transcriptomic comparative analysis reveals a similar pattern of inter- and intra-specific divergences among known (named species with distinct morphology) or suspected cryptic species, species indistinguishable with genetic or morphological data (Table 1). When isolates from the same species are compared, almost all (~ 95%) of the contigs are between 0 and 2% divergent (Figs. 2a-c, 3a and 4, Table 1). Conversely, when isolates from different species are compared, all or almost all (> 99%) of the contig groups fall outside this range (Table 1). The distribution of distances between contigs from different species resembles a normal distribution, which varies in average divergence based on the distance between the species (Figs. 2d and 3b). In all cases, we observed a minority of contigs which were much more divergent than the average, sometimes diverging by more than 50% from each other even when comparing transcriptomes from the same species (Table 1, Additional files 1, 2, 3, 4, 5 and 6: Figures S1–S6). The proportion of these contigs was reduced in analyses comparing only single-copy eukaryotic genes (Additional files 2, 4 and 6: Figures S2, S4 and S6), as opposed to those comparing all matched contigs (Table 1, Additional files 1, 3 and 5: Figures S1, S3 and S5). However, no analysis was completely free of the high-distance contig groups, visible in the graphs as a “tail” of high variation (Table 1, Additional files 1, 2, 3, 4, 5 and 6: Figures S1–S6). Further inspection of these groups, both from all and single copy genes only datasets, showed that problems with alignment, completeness of data and sequencing quality likely account for the majority of the observed high-distance. The general pattern observed in our analysis is useful in demonstrating the utility of transcriptome data for cryptic species diversity studies and identifying potential conversed nuclear barcode markers for microbial eukaryotes (Table 2, Additional file 7: Table S1).

**Table 2:** Average distances of potential barcode markers present in the transcriptomes of all analyzed isolates. Twelve selected potential barcoding markers, identity and their intra- and inter-specific distances

Cluster	Accession #s	Gene name	*C pen*	*C min*	*C pen C min*	*C min C minut*	*E zon* PRA-191	*E zon* PRA-191 LINKS	UK-YT1 RHP1–1
2785	XP_004353437 XP_638491	Signalosome complex protein	0	0	0	7.5	0.1	11.1	0.2
2836	XP_004356442 XP_640858	Proteasome alpha subunit C	0	0	0	11.0	1.7	14.7	0.1
2840	XP_004344004 XP_637130	Phosphoglycerate kinase	0	0	0	13.0	0.4	15.0	0
3192	XP_004368096 XP_642255	Ribulosephosphate 3-epimerase	0.3	0	0.1	12.0	0.4	13.0	0.2
3202	XP_004368095 XP_646606	Proteasome 26S subunit	0	0	0	9.4	0	12.5	0.1
3401	XP_004341728 XP_628938	COP9 signalosome complex	0	0	0	6.3	0.2	14.5	0.2
3510	XP_004341817 XP_646323	Fructose-1,6-bisphosphatase	0	0	0.2	16.3	0.1	14.0	0
3691	XP_004336605 XP_629938	ATP-dependent RNA helicas	0.2	0	0.1	7.8	0	15.0	0
3965	XP_004353478 XP_640508	rRNA PÍseudouridine synthase	0	0.2	0.1	13.2	0.1	12.0	1.2
3986	XP_004336278 XP_628968	Proteasome/cyclosome	0	0	0.02	9.9	0	16.5	0.04
4056	XP_004338975 XP_636972	Proteasome 26S subunit	0	0.04	0.03	11.6	0	14.4	0.03
4574	XP_004335206 XP_643167	NAD-dependent epimerase/dehydratase	0.1	0	0.3	9.6	0.1	15.5	0

Abbreviations: *C pen C. pentatrifurcatum, C min C. minus, C minut C. minutoidum, E zon E. zonatum, UK-YT1* Thecamoebida sp., *RHP1–1* Thecamoebida sp

### Cases of cryptic and inter-species comparisons

#### Cochliopodium

In previous studies, two isolates of *Cochliopodium*, *C. minus* (CCAP 1537/1A) and *C. pentatrifurcatum* (ATCC 30935), were originally described as separate species based on morphological analysis [44, 45]. These isolates were later found to be genetically identical in the commonly used ribosomal and mitochondrial barcoding markers [27, 35]. To further explore the large-scale genetics of these two isolates, we sequenced transcriptomes from both isolates, as well as from *C. minutoidum*, a closely related species, which is clearly distinct in the barcoding markers [27]. From 31,357 *C. minus*, 20,630 *C. pentatrifurcatum*, and 8561 *C. minutoidum* contigs, we extracted 12,767 orthologous subgroups, of which 10,829 had at least one sequence from both *C. pentatrifurcatum* and *C. minus*. This set of groups was filtered to keep only those that were classified as single-copy and which contained at least one identified eukaryotic sequence (Fig. 1). Additionally, we kept only groups that matched one of the 2054 single-copy clusters derived from genomes of *Dictyostelium discoideum* and *Acanthamoeba castellanii* (Fig. 1). This final dataset included 1767 groups, of which 1696 had at least one sequence from both of *C. pentatrifurcatum* and *C. minus* (Fig. 1). Of the 1696 groups, 675 (39.8%) had identical sequences for *C. pentatrifurcatum* and *C. minus*, while an additional 954 groups (53.5%) contained sequences that were no more than 2% divergent between the two species (Fig. 2c). A total of 67 (4.0%) of the contig groups contained sequences with > 2% divergence between *C. pentatrifurcatum* and *C. minus* (Table 1)*.* Further investigation of these groups revealed that the high divergences (sometimes in excess of 50%, Additional files 1 and 2: Figures S1 and S2) were likely the result of grouping errors, i.e. distant paralogs or unrelated genes being grouped together by BLAST. The more moderate divergences were likely due to sequencing error, particularly in the beginning and end of the sequences, or recent paralogs without corresponding orthologs grouping together - a product of either gene loss or, more likely, incomplete transcriptome data. This interspecific distribution is similar to that of the intraspecific comparisons within *C. pentatrifurcatum* and *C. minus*, each of which had 0% intraspecific variation in around 62% of contig groups and < 2% variation in 33–35% of groups, while the proportion of groups with > 2% divergence was around 3–4% (Fig. 2a, b, Table 1). By contrast, in the comparison of *C. pentatrifurcatum* (and also *C. minus –* data not shown) with *C. minutoidum*, no groups were below the 2% divergence cutoff (Table 1); the smallest interspecific distance was 3.8%, while the average was 14.4% and most of the sequences (52.2%) fell between 13 and 18% divergence (Fig. 2d).

#### Endostelium

We compared transcriptomes of *Endostelium zonatum* PRA-191 sequenced by our lab [46] and by another lab [14], as well as a potential new isolate of *Endostelium* denoted as *‘E. zonatum* LINKS’ in the publication of Kang et al. [14]. Of 1292 single-copy eukaryotic contig groups containing sequences of PRA-191 from both labs, 598 (46.3%) were identical between the two transcriptomes, while an additional 642 (49.7%) were less than 2% divergent (Fig. 3a). A total of 52 contig groups (4.0%) had distances greater than 2% (Table 1, Fig. 3a). By contrast, *E. zonatum* LINKS is very divergent, with an average distance of 19.5% from Kang et al. [14] *E. zonatum* PRA-191 (Fig. 3b) and 18.6% from Tekle and Wood [46] *E. zonatum* PRA-191 (data not shown). No contig groups less than 2% divergent were found (Table 1, Fig. 3b). This distribution is more similar to the comparison of *C. pentatrifurcatum/minus* to *C. minutoidum* than to any of the intraspecific comparisons (Table 1, Fig. 2), indicating that this isolate likely is not *E. zonatum*, but instead is probably a new *Endostelium* species.

### Isolates of Thecamoebida

While comparing the transcriptome data of various Thecamoebida species in attempt to place a new isolate in the Thecamoebida tree (Melton et al. in press), we noticed that many sequences from our new isolate were very similar or identical to sequences published by Kang et al. [14] for their isolate Thecamoebida RHP1–1. To further explore this similarity, we compared these two transcriptomes using our pipeline. We were able to match 5739 contig groups, 1621 of which were verified as likely single-copy eukaryotic groups due to matching the single-copy clusters from the amoebozoan genomes. A total of 801 contig groups (49.4%) show no divergence between the two isolates, while 752 additional groups (46.4%) have a distance of less than 2% (Fig. 4). A total of 68 of the contig groups (4.2%) were greater than 2% divergent between the two isolates (Table 1, Fig. 4). The distribution of these groups appears very similar to other intra-species comparisons (Figs. 2a, b, c and 3a), indicating that the isolates are likely the same species.

### Identification of potential barcode markers from transcriptome data

We used criteria including pattern of sequence divergence (COI-like), ubiquity, nature of paralogy and evolutionary conservation to choose potential barcode markers among transcriptome contigs analyzed. A total of 660 clusters were present in at least 2 of the amoebozoan clades analyzed, of which 217 were present in all three clades (Additional file 7: Table S1). A total of 41 clusters were present in every transcriptome analyzed; 12 of these well-described groups (genes) are presented along with their gene IDs, names and accession numbers (Table 2). The selected markers have different evolutionary rates and thus may be suitable for different levels of taxonomic delineation. All of these markers delineated all species analyzed in a consistent manner.

## Discussion

### Transcriptome data: an appropriate tool for cryptic species diversity study

The transcriptome is an ideal source of data for evolutionary studies that rely on highly conserved and orthologous markers. Evolutionary studies in microbial eukaryotes have lagged behind compared to other macrobial organisms partly due to limited genetic data. Microbes are often difficult to grow (yielding insufficient DNA for PCR) and require several trials to amplify single gene products since most of the universal primers designed for multicellular eukaryotes fail to work in most microbes [5]. For this reason and due to the recent advances in HTS, the cost of acquiring transcriptome data from difficult microbes (e.g. [47]) is becoming comparable to obtaining single markers using Sanger sequencing methods [48]. The feasibility of obtaining large amounts of genetic data from small amounts of starting material is making transcriptomics a method of choice in the evolutionary study of microbes. Thus, transcriptome data from underrepresented and unculturable microbes has been growing exponentially in the last decade [49, 50]. This data has been mostly used for inferring deep phylogenetic history [14, 42]. In this study, we demonstrate that transcriptome data is also appropriate for understanding cryptic species diversity in microbial eukaryotes.

One of the anticipated challenges in comparative transcriptomic study in cryptic or closely related species is that the results might vary based on the physiological state of an organism at the time of RNA collection [49]. Moreover, individuals may express different variants of a gene (paralog) depending on the developmental phases or other environmental factors, which could affect comparative study at lower taxonomic or cryptic levels. Similarly, data quality and sequencing error [51] might affect species divergence calculations. We developed a bioinformatics pipeline that is suited to handle most of these concerns through stepwise data quality control and tree-based paralog sorting (Wood and Tekle in prep.). Analyses of transcriptome data from suspected or known cryptic species using our pipeline yielded consistent results regardless of the methods of culturing, RNA collection or sequencing. Using our approach we were able to resolve discrepancies between morphology and mitochondrial genes in an amoeboid microbe [27], uncover misidentification in previous published work [14], and discover a new species (Melton et al. in press).

Distance calculations of matched groups from whole transcriptome data show an interesting pattern among isolates of the same species originating from different labs, as well as genetically similar (suspected cryptic) species. Divergences exceeding 2% between these isolates and species accounted for comparable proportions of the contigs (5.89–9.31%, Table 1). These proportions decreased (2.91–4.87%) when only single copy genes were considered in our analyses (Table 1). Closer inspections of the high-distance groups (> 2%) indicate a number of possible explanations including mismatched groups, sequencing error and methodological limitation. In some cases high divergences resulted when genes without their orthologous counterparts across strains (due to the incomplete nature of transcriptome data) are mismatched with distant gene families or paralogs. While most the sequencing errors were greatly reduced by trimming the beginning and ending of the sequences, in rare cases sequencing error (low data quality) was observed to contribute to the observed high distances. Similarly, high divergences were observed as a result of unrelated or very distant genes grouping together erroneously as a result of a short overlap/s in aligned sequences. Therefore, the high distances observed in our analyses in this minority of groups are likely not indicative of actual divergence or speciation. On the contrary, high divergences exceeding the species delimitation threshold (> 2%) have been reported in the COI (e.g. [52]) and ribosomal genes [29]). This has been one of the major criticisms for the universal use of these markers for DNA based barcoding [33]. Our study shows that with improved sequencing and analytical approaches, transcriptomes offer a multitude of data that can be used for comprehensive comparative analysis of cryptic diversity. Transcriptome data also has an added advantage in that the large genetic data can be concatenated to reconstruct species tree, which could server as corroborating evidence as has been used in other similar HTS studies based on genome data [53].

### Selection of appropriate barcode markers in microbial eukaryotes

Given the idiosyncratic nature of DNA barcoding, transcriptome data provide an opportunity to explore many genetic makers that can be appropriately applied to different lineages with varying evolutionary rates and history. In this study, we identified 660 single copy markers in amoeboid microbes based on evolutionary rate and ubiquity. All these markers have intraspecific divergences below 2%, while also includes a range of interspecific divergences that can be applied specifically for a single genus or for multiple genera or clades. We present 12 highly conserved markers found in the three diverse lineages of amoeboid microbes examined in this study (Table 2). These markers are involved in important biological pathways such as glucose metabolism (see Table 2). All these markers were able to distinguish the species analyzed in this study in a consistent manner similar to other barcode markers [26, 27, 54]. The selected markers also provide a range of distances at the interspecific level. This is important for delineation of recently diverged species falling close to or within the threshold values for species delimitation [35]. Defining a barcode gap based on single marker is a controversial subject due to the variations that exist in evolutionary rates among lineages [32]. Transcriptome data as whole or selected barcode markers with varying evolutionary rates will enable a more comprehensive assessment and eliminate the dependency on fixed delimitation thresholds.

### Taxonomic notes

Amoeboid eukaryotes belonging to the supergroup Amoebozoa include diverse lineages that are largely understudied. In the last 3 years the supergroup has seen an explosion of transcriptome data mostly used to study deep relationships within the supergroup [11, 14, 46, 55]. Both previous and more recent molecular studies have revealed some major discordances with the morphology based classification system at both lower and higher taxonomic levels [14, 25, 47]. Hence, the Amoebozoa provide an ideal system to test the utility of transcriptome data in resolving discordances related to cryptic diversity. In this study, we present results of two major discordances: different morphologies with the same genetics (*Cochliopodium*) and similar morphologies with different genetics (*Endostelium*).

*Cochliopodium* is a genus of lens-shaped amoeba in which taxonomy has greatly relied upon the morphology of flexible microscales present in the cell coat [56, 57]. Recent studies have reported that some species of *Cochliopodium* with dramatically different scale morphology have identical COI and SSU-rDNA gene sequences [27, 35]. This discordance created great confusion in the taxonomy of the genus since most of its members had been identified by the elaborate scale morphology they display [35]. In this study, we used large-scale transcriptome data to reliably show that the two lineages (*Cochliopodium pentatrifurcatum and C. minus*) are the same species despite their drastic difference in scale morphology [44, 45]. Scale morphology in *Cochliopodium* is made of proteinaceous material and is encoded in the genome [58]. It is not clear why different populations belonging to the same species would express different scale morphologies. However, phenomena such as temporal or environmental factors might control the expression of scale morphology in amoebae*.* Some *Cochliopodium* species have even been observed to possess two types of scales in one individual (personal comm. Eckhard Völcker). Similarly, Amoebae belonging to the genus *Korotnevella* have also been reported to sometimes express more than one type of scale [59]. The *Cochliopodium* isolates (*C. pentatrifurcatum* and *C. minus*) originated from different localities but were grown under similar culturing conditions in our laboratory, so environmental factors are unlikely to explain the difference in scale morphology in these species. Further investigation is needed to examine the factors affecting expression of different scale morphologies in *Cochliopodium* and other amoebae*.* Given the overwhelming genetic evidence and the unreliability of scale morphology in the genus, we recommend the synonymization of *Cochliopodium pentatrifurcatum* to *C. minus* based on taxonomic priority. Similarly, using the same approach we discovered that two undescribed isolates of amoebae belonging to clade Thecamoebida from our lab and the publication of Kang et al. [14] are conspecific. Our isolate is currently being described as a new genus of Thecamoebida (Melton et al. in press).

A second case of discordance exemplifying cryptic species problems is an amoeba belonging to the genus *Endostelium.* Kang et al. [14] published transcriptome data of an isolate designated as *‘E. zonatum* LINKS’ in their phylogenomic study. Comparison of this isolate with our and their *E. zonatum* PRA-191 showed that the two isolates (LINKS and PRA-191) are very divergent (average 19.5%), far beyond the species delimitation thresholds used in any organism (Tables 1 and 2). On the contrary, the two strains of *E. zonatum* PRA-191 sequenced in two different labs had similar divergences to those observed between the isolates of the same species (Tables 1 and 2). Based on our finding, the isolate designated as *‘E. zonatum* LINKS’ is a typical case of the cryptic species problem and should be renamed or described as new species.

## Conclusion

Our study illustrates the practical applications of transcriptome data in resolving cryptic diversity problems and other forms of discordance that exist between molecular and morphological data in microbial eukaryotes. The transcriptome can also play a role in exploration of biodiversity and discovery of new species. The approach used in this study is applicable to non-microbial eukaryotes and other sources of genetic data.

## Materials and methods

### Transcriptome data collection and assembly

Transcriptomes of *Cochliopodium pentatrifurcatum* (ATCC® 30,935™), *Cochliopodium minus* (CCAP 1537/1A), and *Endostelium zonatum* (PRA-191) from previous studies [46, 60] were used. Additional transcriptomes of *Endostelium zonatum* PRA-191 (SRX2163157), *Endostelium zonatum* LINKS (SRX2691243), and Thecamoebida isolate RHP1–1 (SRX2691210) from Kang et al. [14] were also retrieved from NCBI. We also sequenced new transcriptomes of *Cochliopodium minutoidum* (CCAP 1537/7) and a new Thecamoebida isolate designated as UK-YT1 (Melton et al. in press). New transcriptome data were collected using the same protocol, for total RNA, as in [60].

FastQC (http://www.bioinformatics.babraham.ac.uk/projects/fastqc/) was used to inspect reads from Kang et al. [14] and from our newly sequenced transcriptomes for quality and length. Illumina adaptor sequences and low quality reads with score below 30 were removed using BBDuk (Joint Genome Institute, U.S. Department of Energy, Walnut Creek, CA USA). The trimming of low quality reads from both ends (“rl” trim mode) is based on Phred algorithm implemented in BBDuk. Using the same program we also removed reads shorter than 60 bp after trimming. The remaining reads were assembled de novo using rnaSPAdes-version 0.1.1 [61] with default parameters. The resulting contigs were then filtered with custom Python scripts to remove those less than 300 bp in length. Contigs were then separated into ribosomal, bacterial, eukaryotic, and unidentified contigs using BLAST [62] and USEARCH [63] against databases of RefSeq ribosomal, prokaryotic, and eukaryotic sequences. TransDecoder (http://transdecoder.sf.net) was then run on the eukaryotic and unidentified contigs from each transcriptome to trim non-coding regions and collect the resulting CDS’s, which were then used for the subsequent transcriptome-wide comparisons.

### Ortholog finding and divergence calculation with the species comparison pipeline

**Fig. 5:**
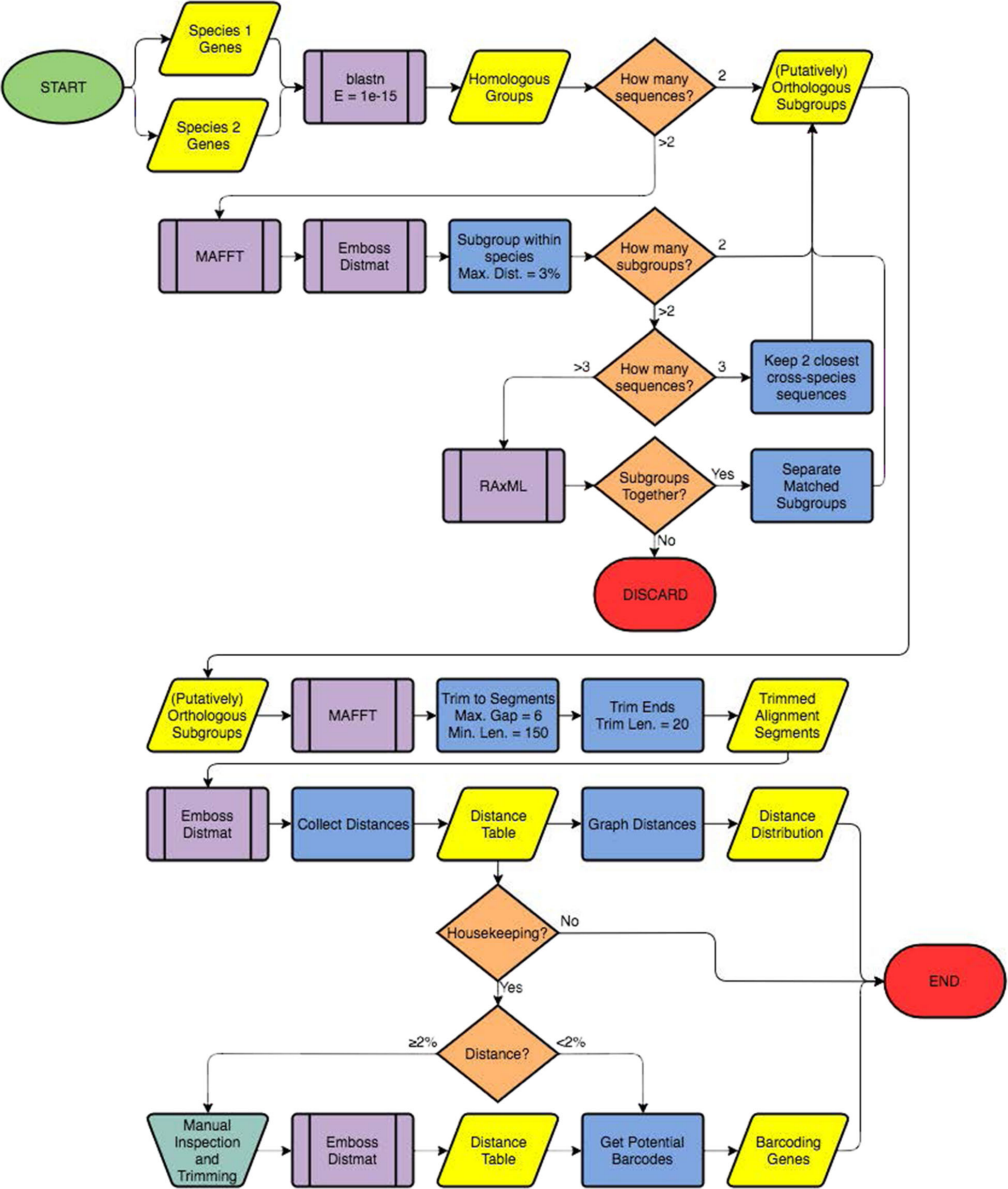
Flowchart of automated species comparison pipeline

We designed an automated pipeline using BLAST [62], MAFFT [64], EMBOSS [65], RAxML [66, 67] and Biopython to find orthologous contigs between assembled and filtered transcriptome sequences, then align them and calculate divergence. A flowchart outlining steps of the species comparison pipeline is presented in Fig. 5. The pipeline is available from the authors upon request. In the first step of the pipeline, an all-vs.-all BLAST is conducted on the transcriptome contigs, and matching contigs with an e-value lower than 1e-15 are clustered into homologous groups (HGs). In the second step, any multicopy HGs are separated into putative orthologs, as follows: If more than one sequence from a given transcriptome is present in a HG, the sequences in that HG are aligned using MAFFT [64] and a distance matrix is calculated using EMBOSS’s distmat function [65]. Sequences from within each transcriptome are subdivided based on distance from each other with a maximum distance cutoff of 2%, which corresponds to the approximate barcoding gap in *Cochliopodium* [27]. The 2% threshold is based on analysis of COI gene involving a large taxon sampling of the genus *Cochliopodium* [27]. In this genus maximum intraspecific (0.9%) and minimum interspecific (2.8%) divergences are recorded, which put the barcode gap approximately at 2% (see [27]). The recommended threshold is also applicable to other amoebozoans [28, 29]. This threshold is primarily used to sort paralogs, identify and examine divergent sequences in intrastrain and HG comparisons. The selected threshold allows contigs with very little variation or overlap to still be grouped together if they come from the same gene. Gene trees are then built using RAxML [66] to match each within-transcriptome subgroup to its orthologs in the other transcriptomes in the dataset, generating the final Orthologous SubGroups (OSGs). In cases where there are only three sequences in a HG (preventing gene tree building with RAxML) and subdivision is required - i.e. two sequences are present from one transcriptome and one is present from another transcriptome - the less distant of the two paralogs from one transcriptome is matched with the single sequence from the other transcriptome, and the more distant sequence is removed (Fig. 5). This step saves all OSGs to a single folder, while also separating all single-copy HGs (also putative orthologs) to a separate location for further downstream analysis. In the final step of the Pipeline, distance matrices for each OSG are generated and collected into a single spreadsheet for further analysis. To accomplish this, each OSG is aligned using MAFFT with default settings, before and after trimming sequences to remove poor-quality regions. Distance matrices for each OSG are generating using EMBOSS’s distmat function, measuring uncorrected p-distance. The minimum, maximum, and average intra- and inter-species distances within each OSG are collected from the distance matrices into the final spreadsheet.

The above pipeline was used to find OSGs in the following groups: *Cochliopodium* (*C. pentatrifurcatum* ATCC 30935 vs. *C. minus* CCAP 1537/1A (transcriptomes from two independent samples each) vs. *C. minutoidum* CCAP 1537/7), *Endostelium* (*E. zonatum* PRA-191 Tekle and Wood 2017 [46] vs. *E. zonatum* PRA-191 Kang et al. [14] vs. *E. zonatum* LINKS [14]), and Thecamoebida (undescribed UK-YT1 vs. Thecamoebida RHP1–1 [14] isolates). On average it takes about four hours to run our pipeline on a pair of species in a regular desktop computer with 32 GB memory.

### Generation of distance histograms and identification of potential barcodes

Distances within OSGs generated by the pipeline were organized into three partitions; all OSGs, only single-copy OSGs (i.e. HGs which needed no subdividing) containing identified eukaryotic contigs, and only OSGs from the above which matched a set of 2054 single-copy genes shared between the genomes of *Dictyostelium discoideum* and *Acanthamoeba castellanii.* This set of 2054 genes was extracted from comparing the genomes with OrthoVenn [43], a web application for comparing and clustering the genes in various eukaryotic and prokaryotic genomes. Matches were removed if they were not unique; that is, if more than one OSG matched to a single OrthoVenn genome cluster. Single copy genes (orthologs) are ideal for species comparison study since matches between non-orthologs contigs might occur in multicopy genes (paralogs) due to the incomplete nature of transcriptome data. Distances from each of the three partitions were analyzed and histograms of distance vs. number of contigs were generated in Excel.

Potential barcodes were selected from the single-copy, OrthoVenn cluster-matching OSGs if their intraspecific distances in *C. pentatrifurcatum*, *C. minus*, and *E. zonatum* PRA-191 were less than 2%, where they exist. OSGs from each comparison were combined based on their matching OrthoVenn cluster, and clusters were kept only if at least two genera (from *Cochliopodium, Endostelium,* and Thecamoebida, above) were represented. Clusters with sequences from all analyzed transcriptomes were examined by BLAST and accession number of genome sequences were used to determine gene identity.

## Additional files

Additional file 1: **Figure S1.** Histogram of average distances within ortholog groups, including single- and multi-copy identified and unidentified groups, from genus *Cochliopodium*. A. *C. pentatrifurcatum* intraspecific variation. B. *C. minus* intraspecific variation. C. *C. pentatrifurcatum*-*C. minus* inter-specific distance. D. *C. pentatrifurcatum*-*C. minutoidum* inter-specific distance. (PDF 88 kb)

Additional file 2: **Figure S2.** Histogram of average distances within genome-matching and nonmatching single-copy eukaryotic ortholog groups from genus *Cochliopodium*. A. *C. pentatrifurcatum* intraspecific variation. B. *C. minus* intraspecific variation. C. *C. pentatrifurcatum*-*C. minus* inter-specific distance. D. *C. pentatrifurcatum*-*C. minutoidum* interspecific distance. (PDF 88 kb)

Additional file 3: **Figure S3.** Histogram of average distances within ortholog groups, including single- and multi-copy identified and unidentified groups, from genus *Endostelium*. A. *E. zonatum* PRA-191 transcriptomes sequenced in different labs [14, 46]. B. *E. zonatum* PRA-191 sequenced by Kang et al. [14] vs. *‘E. zonatum’* LINKS sequenced by Kang et al. [14]. (PDF 76 kb)

Additional file 4: **Figure S4.** Histogram of average distances within genome-matching and nonmatching single-copy eukaryotic ortholog groups from the genus *Endostelium*. A. *E. zonatum* PRA-191 transcriptomes sequenced in different labs (Tekle and Wood 2017 [46]). B. *E. zonatum* PRA-191 sequenced by Tekle and Wood 2017 [46] vs. *‘E. zonatum’* LINKS sequenced by Kang et al. [14]. (PDF 75 kb)

Additional file 5: **Figure S5.** Histogram of average distances within ortholog groups, including single- and multi-copy identified and unidentified groups, from comparison between isolate undescribed UK-YT1 and Thecamoebida sp. RHP1–1 [14]. (PDF 24 kb)

Additional file 6: **Figure S6.** Histogram of average distances within genome-matching and nonmatching single-copy eukaryotic ortholog groups from comparison between isolate undescribed UK-YT1 and Thecamoebida sp. RHP1–1 [14]. (PDF 25 kb)

Additional file 7: **Table S1.** Intra- and inter-specific distances for all potential barcoding genes obtained from transcriptome data. (XLSX 105 kb)

## Abbreviations

ATCC: American Type Culture Collection
BLAST: Basic local alignment search tool
CCAP: Culture Collection of Algae and Protozoa
mtDNA: Mitochondrial DNA
Refseq: Reference sequence
RNAseq: Ribonucleic acid sequencing
