# Supplementary figures and images for: A practical implementation of large transcriptomic data analysis to resolve cryptic species diversity problems in microbial eukaryotes
Source: BMC Evol Biol. 2018 Nov 16;18:170. doi: 10.1186/s12862-018-1283-1 (PMC6240226; doi:10.1186/s12862-018-1283-1)

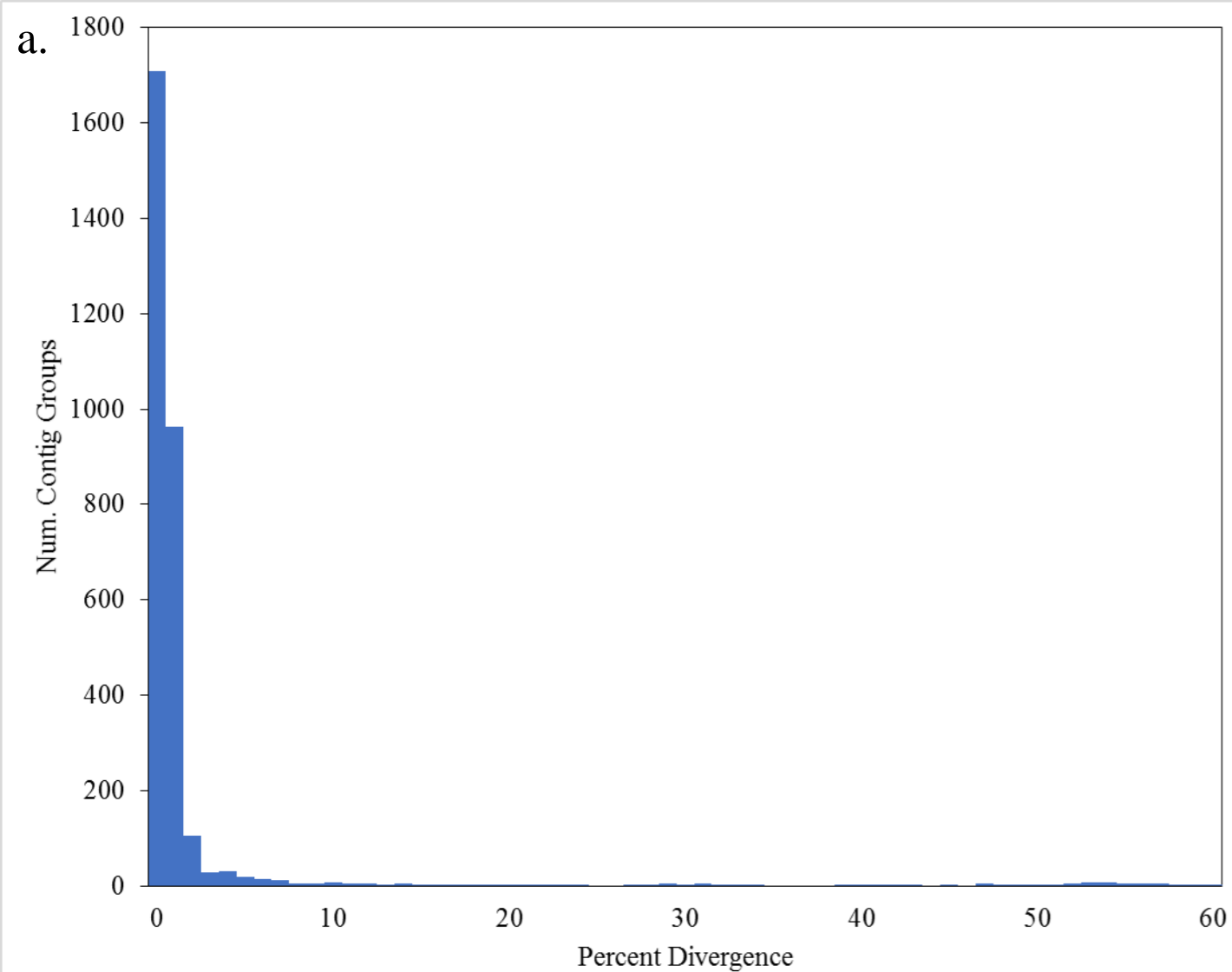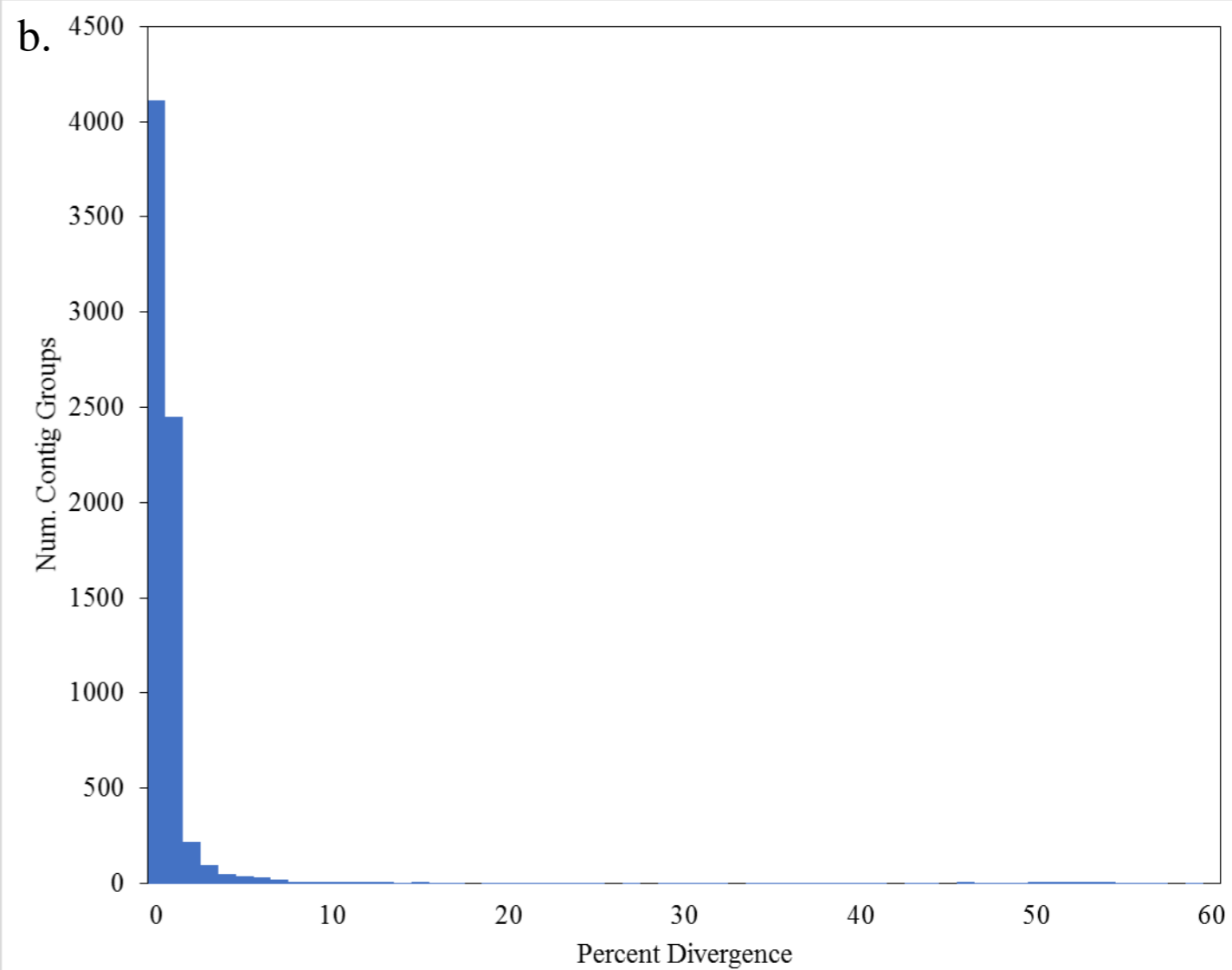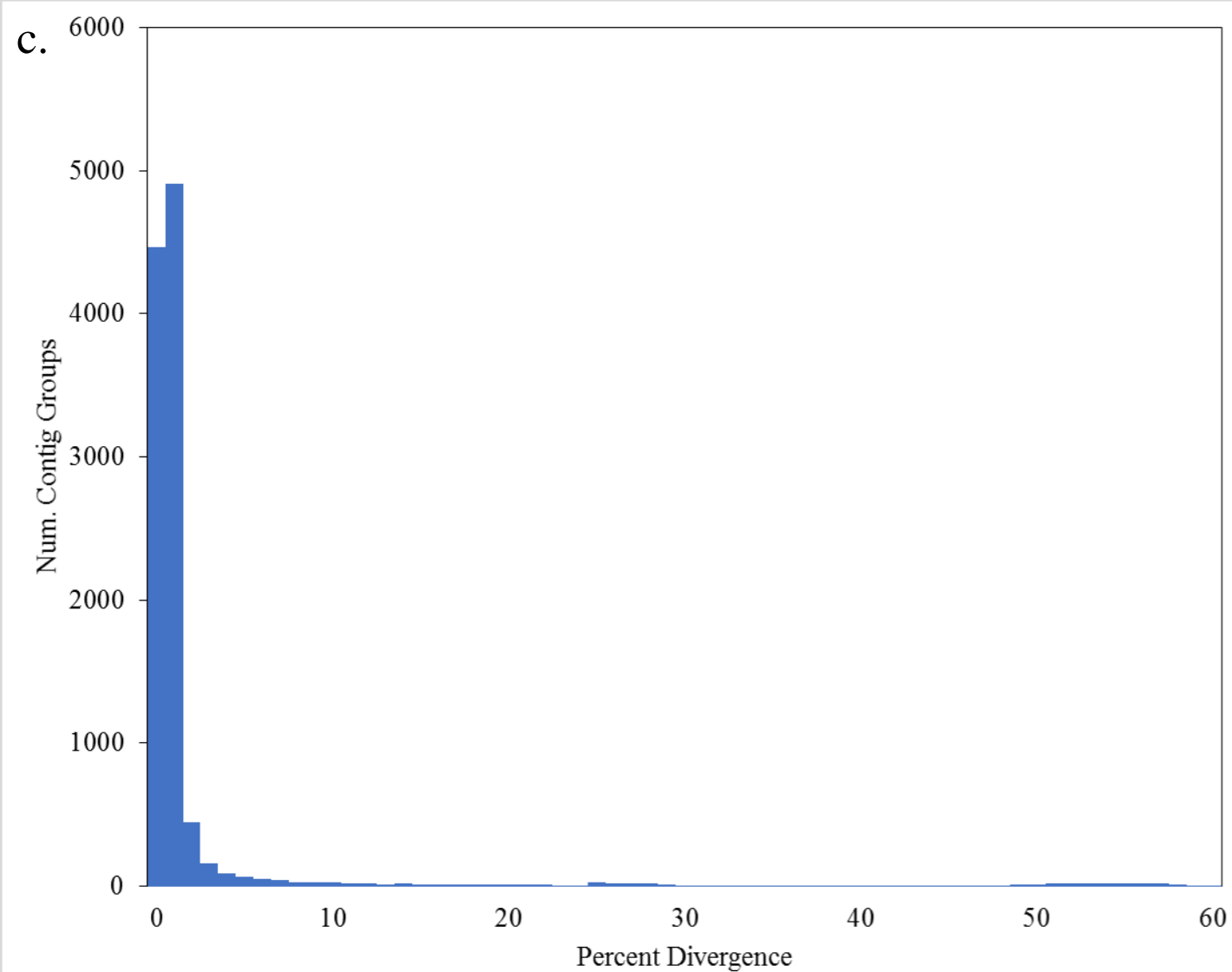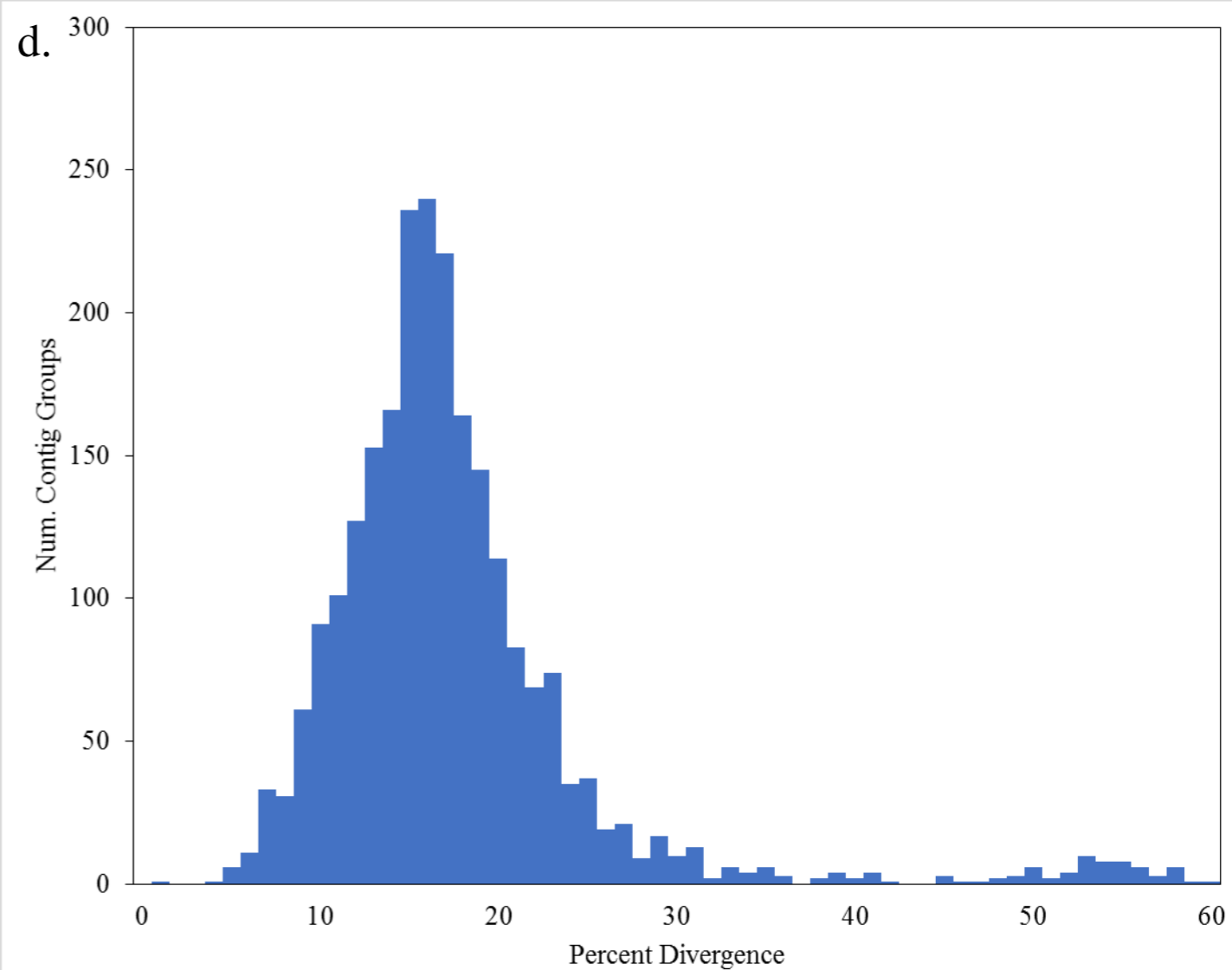

Supplement: Supplementary file 1 — Figure S1. Histogram of average distances within ortholog groups, including single- and multi-copy identified and unidentified groups, from genus Cochliopodium. A. C. pentatrifurcatum intraspecific variation. B. C. minus intraspecific variation. C. C. pentatrifurcatum-C. minus inter-specific distance. D. C. pentatrifurcatum-C. minutoidum inter-specific distance. (PDF 88 kb) [file 12862_2018_1283_MOESM1_ESM.pdf]

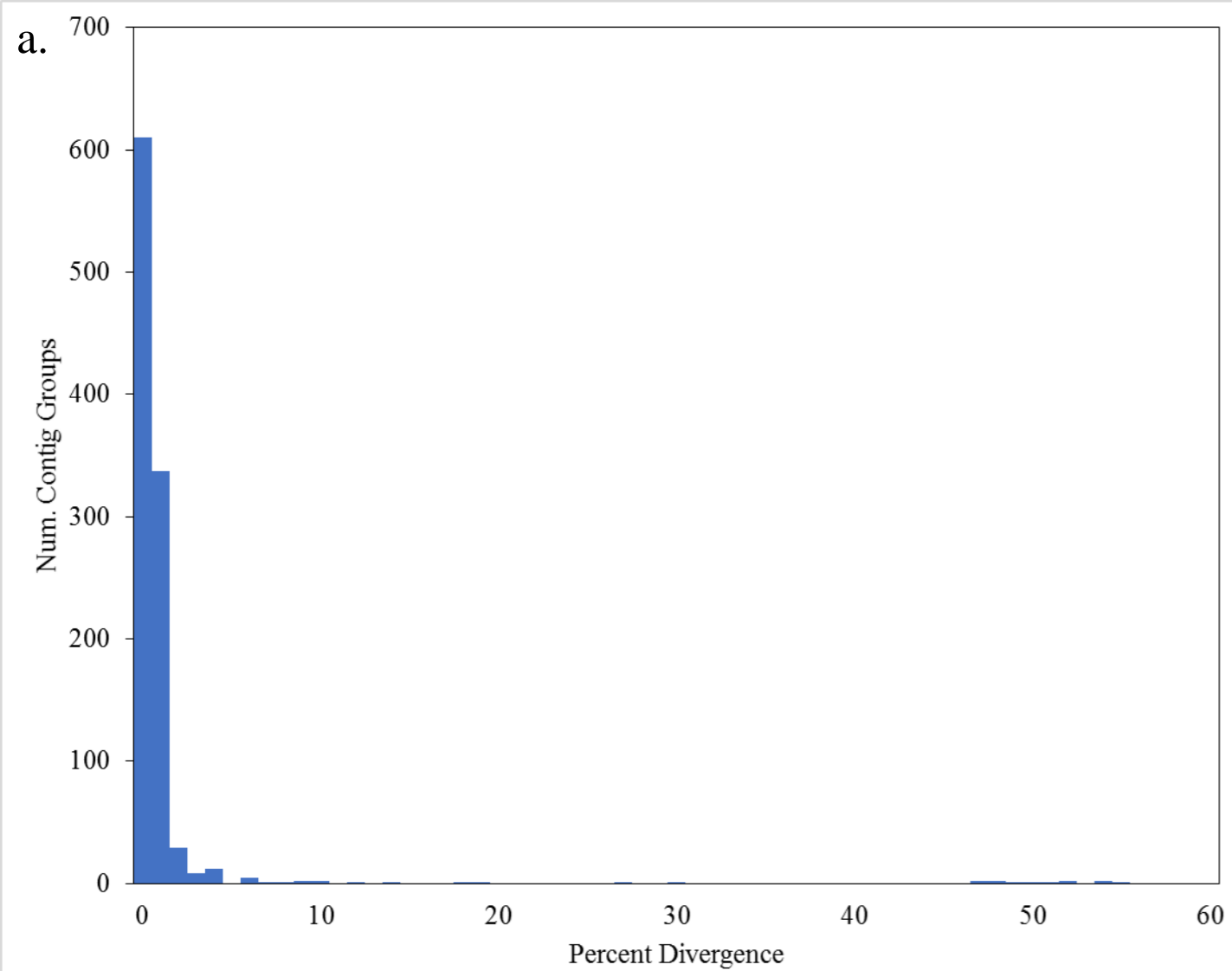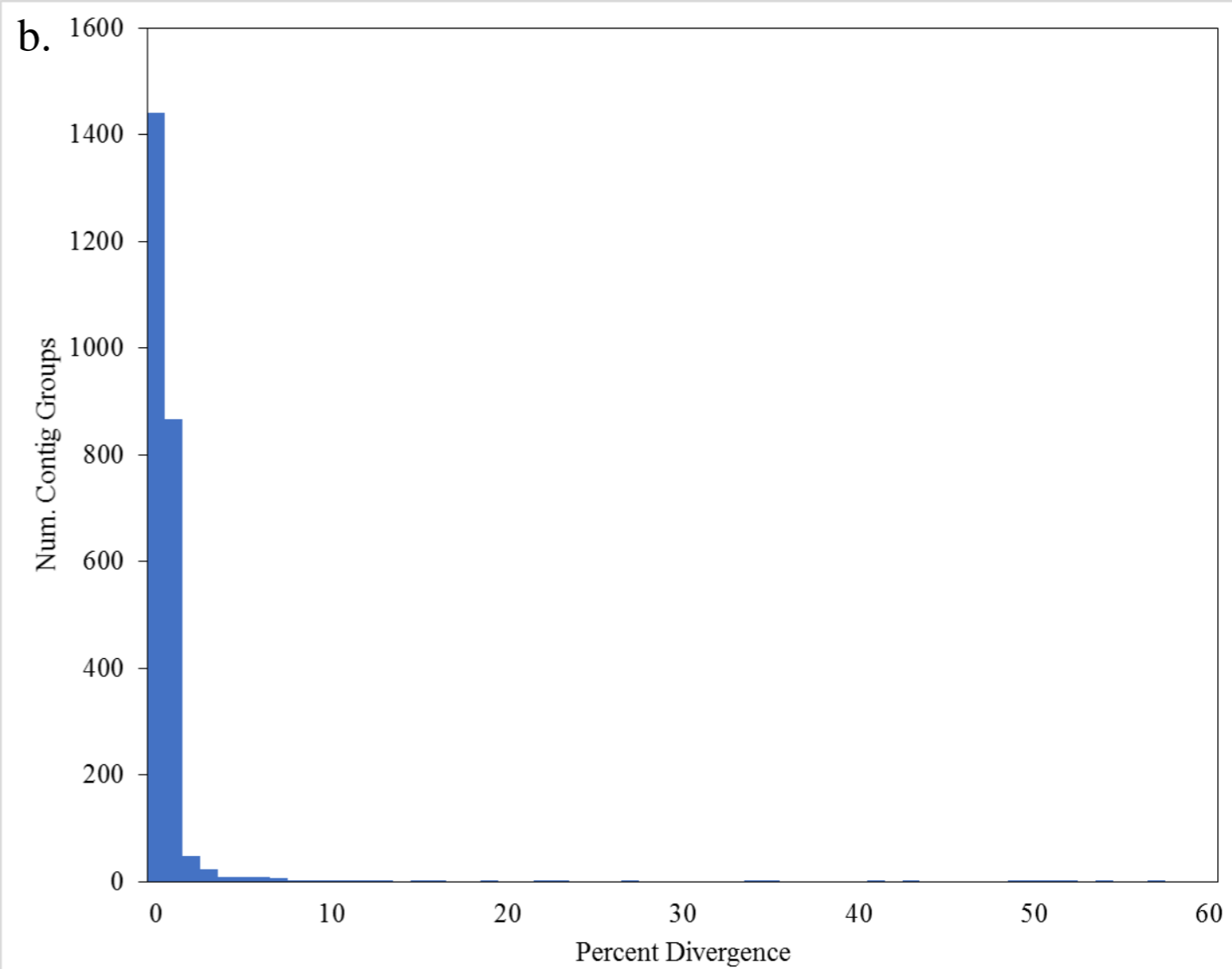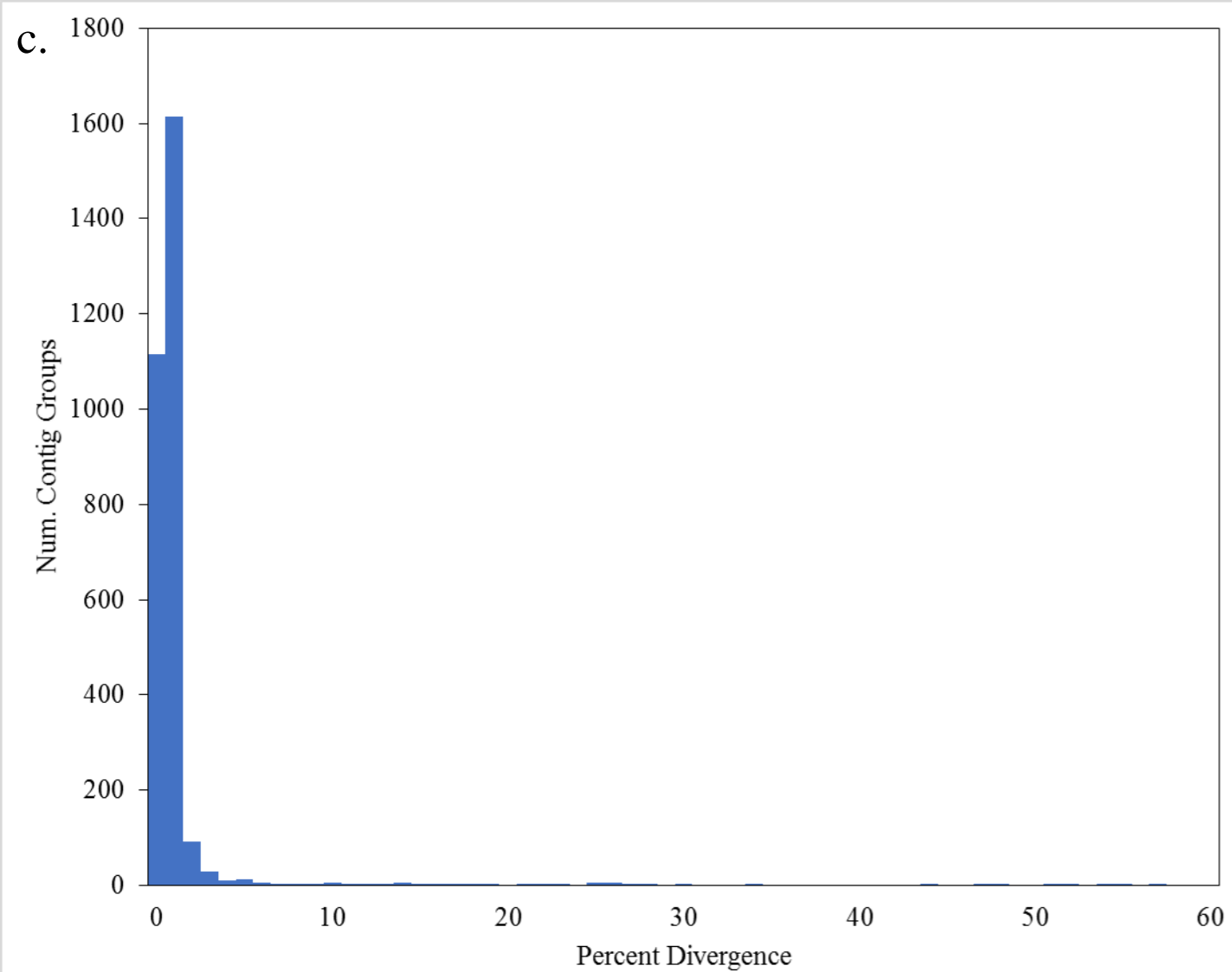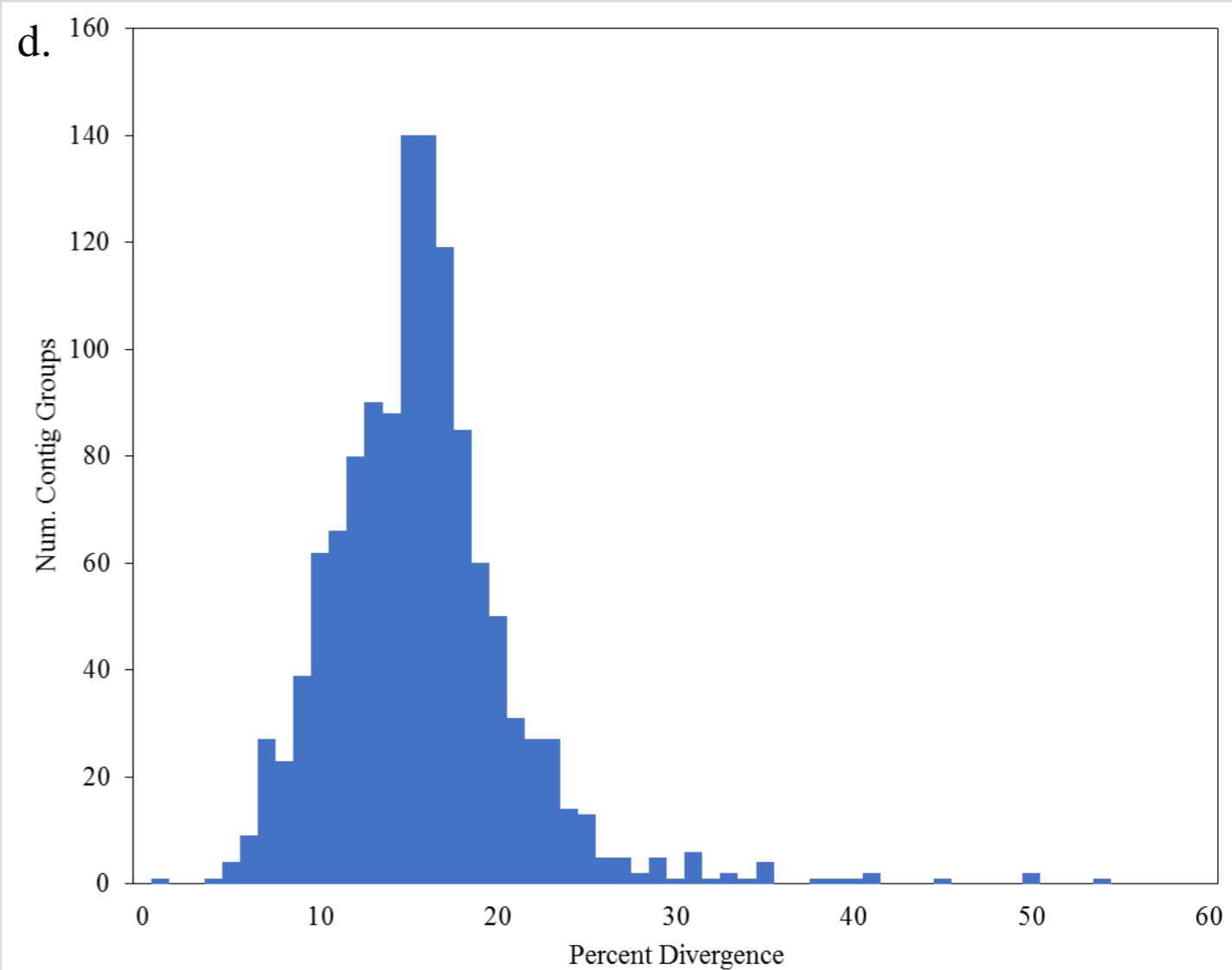

Supplement: Supplementary file 2 — Figure S2. Histogram of average distances within genome-matching and nonmatching single-copy eukaryotic ortholog groups from genus Cochliopodium. A. C. pentatrifurcatum intraspecific variation. B. C. minus intraspecific variation. C. C. pentatrifurcatum-C. minus inter-specific distance. D. C. pentatrifurcatum-C. minutoidum interspecific distance. (PDF 88 kb) [file 12862_2018_1283_MOESM2_ESM.pdf]

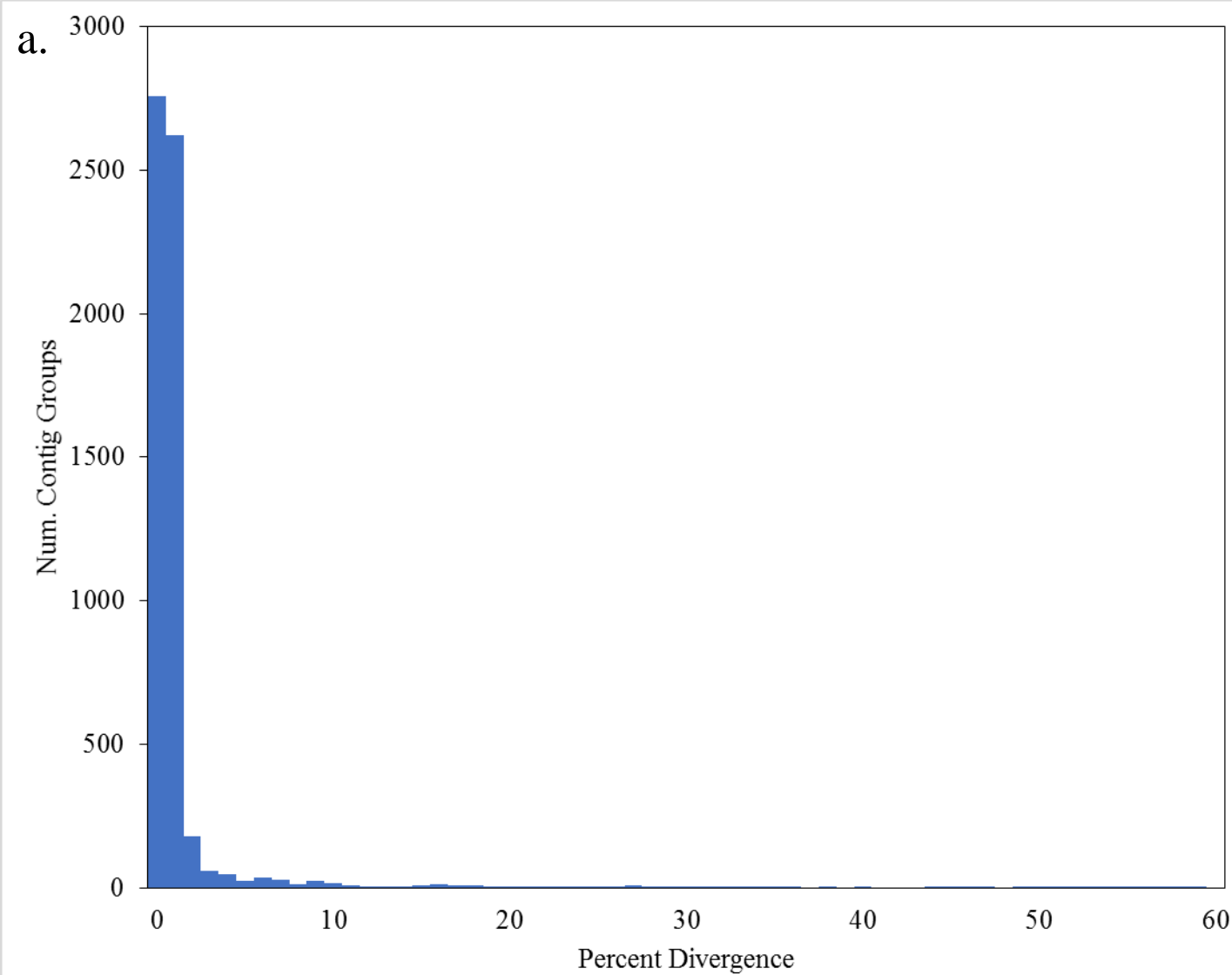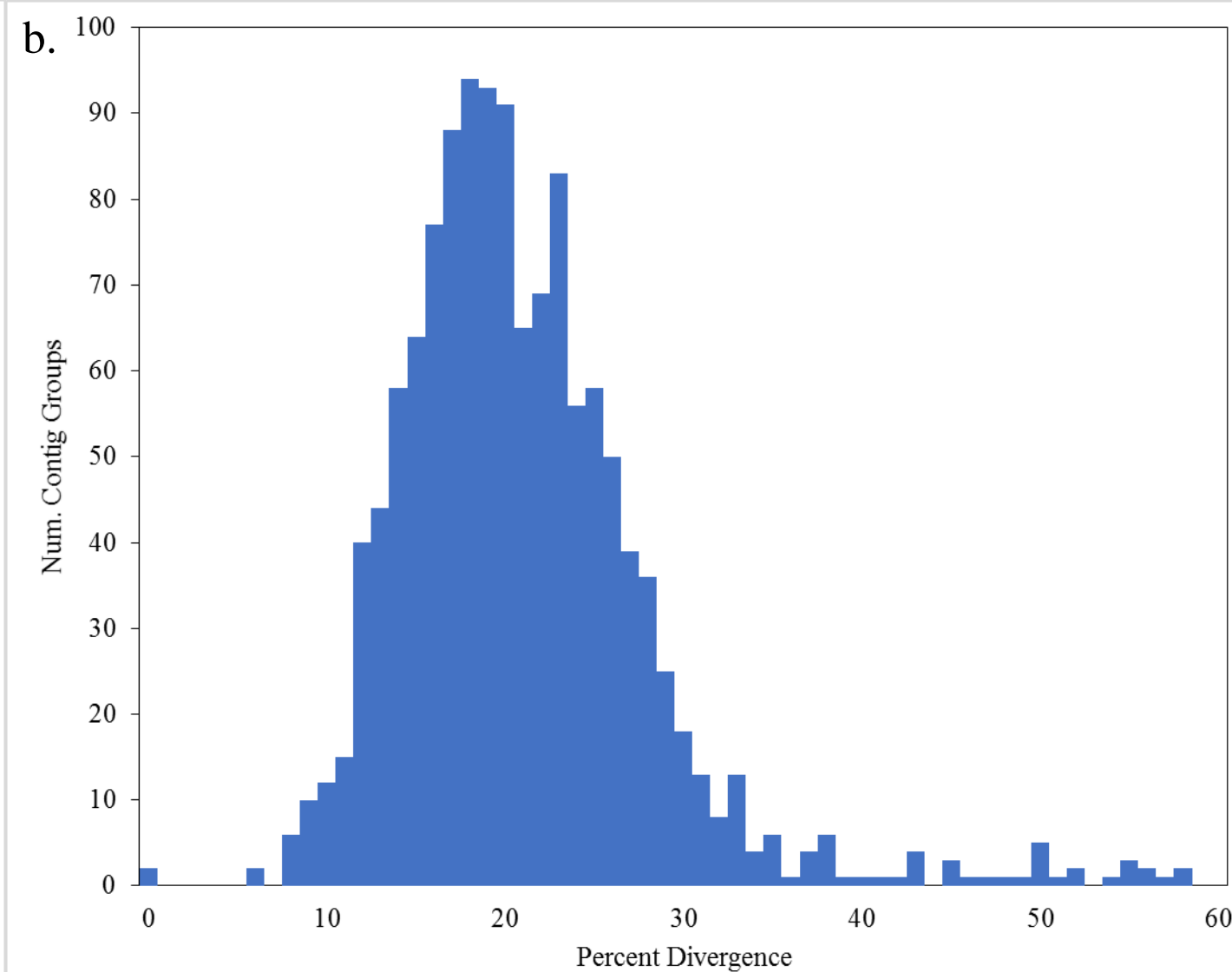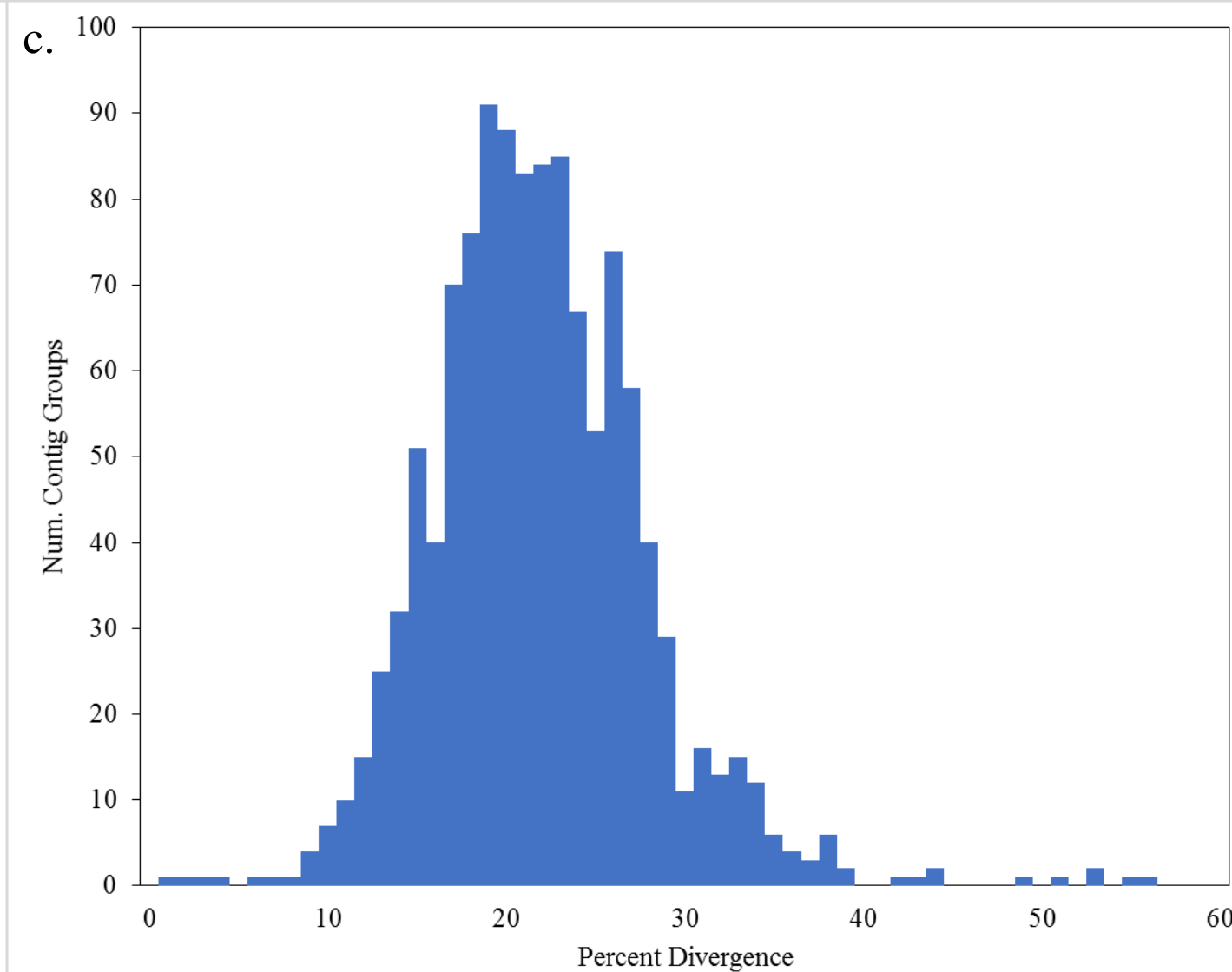

Supplement: Supplementary file 3 — Figure S3. Histogram of average distances within ortholog groups, including single- and multi-copy identified and unidentified groups, from genus Endostelium. A. E. zonatum PRA-191 transcriptomes sequenced in different labs [14, 46]. B. E. zonatum PRA-191 sequenced by Kang et al. [14] vs. ‘E. zonatum’ LINKS sequenced by Kang et al. [14]. (PDF 76 kb) [file 12862_2018_1283_MOESM3_ESM.pdf]

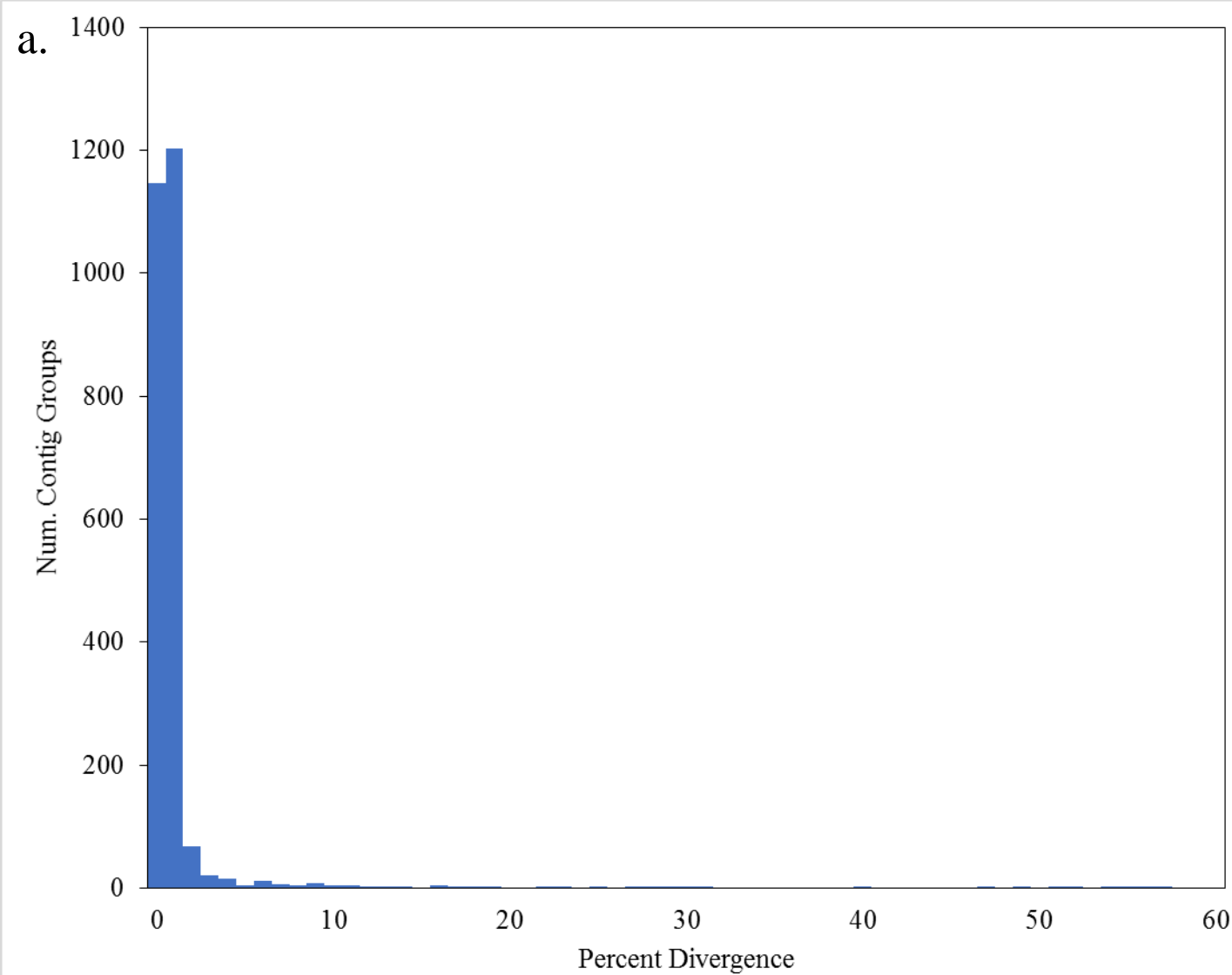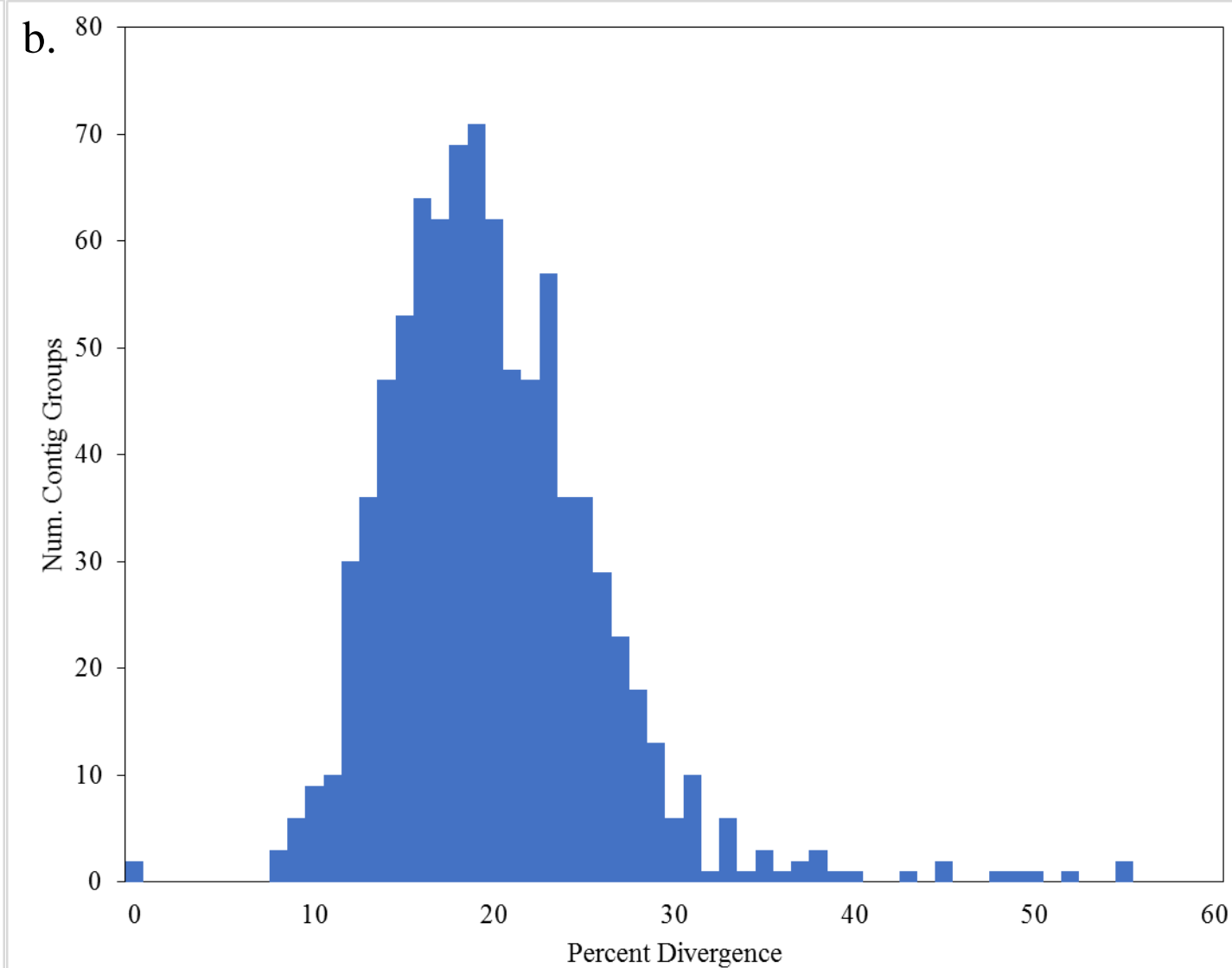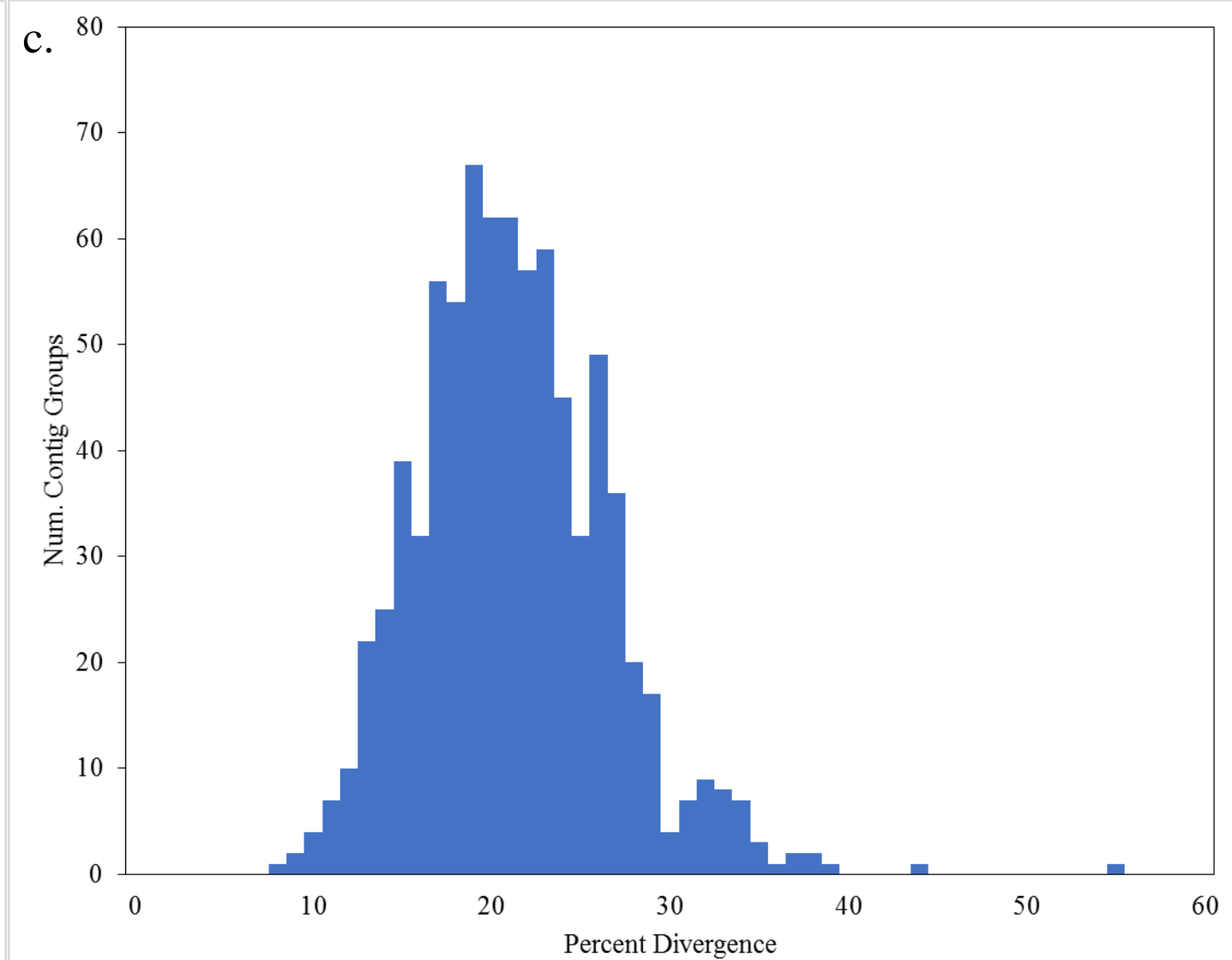

Supplement: Supplementary file 4 — Figure S4. Histogram of average distances within genome-matching and nonmatching single-copy eukaryotic ortholog groups from the genus Endostelium. A. E. zonatum PRA-191 transcriptomes sequenced in different labs (Tekle and Wood 2017 [46]). B. E. zonatum PRA-191 sequenced by Tekle and Wood 2017 [46] vs. ‘E. zonatum’ LINKS sequenced by Kang et al. [14]. (PDF 75 kb) [file 12862_2018_1283_MOESM4_ESM.pdf]

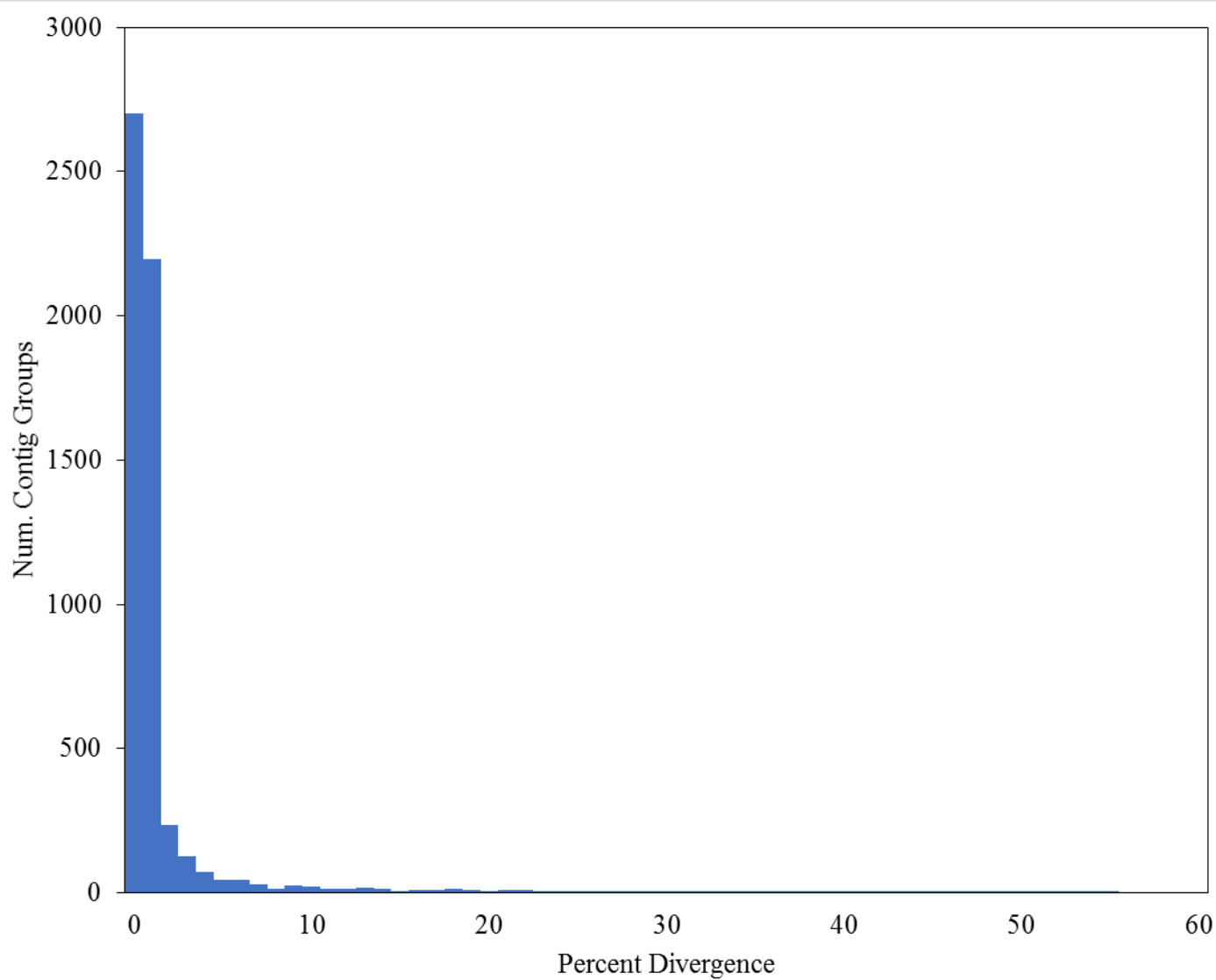

Supplement: Supplementary file 5 — Figure S5. Histogram of average distances within ortholog groups, including single- and multi-copy identified and unidentified groups, from comparison between isolate undescribed UK-YT1 and Thecamoebida sp. RHP1–1 [14]. (PDF 24 kb) [file 12862_2018_1283_MOESM5_ESM.pdf]

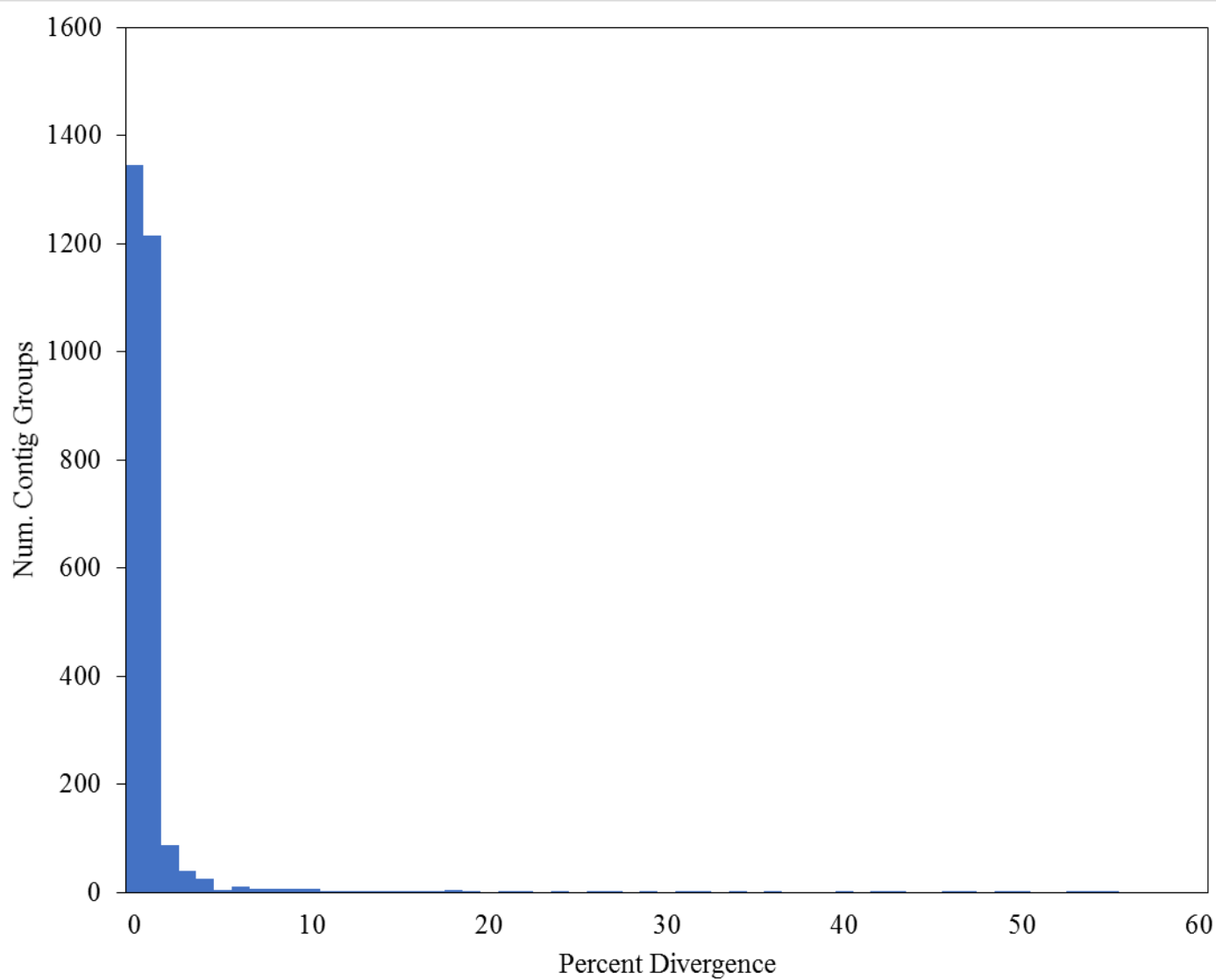

Supplement: Supplementary file 6 — Figure S6. Histogram of average distances within genome-matching and nonmatching single-copy eukaryotic ortholog groups from comparison between isolate undescribed UK-YT1 and Thecamoebida sp. RHP1–1 [14]. (PDF 25 kb) [file 12862_2018_1283_MOESM6_ESM.pdf]
